# Editorial: Pavlovian-instrumental transfer: Neurobehavioral and clinical findings

**DOI:** 10.3389/fnbeh.2023.1151180

**Published:** 2023-02-20

**Authors:** Vincent D. Campese, Vincent Laurent

**Affiliations:** ^1^Department of Psychology and Behavioral Sciences, University of Evansville, Evansville, IN, United States; ^2^Faculty of Science, School of Psychology, University of New South Wales, Sydney, NSW, Australia

**Keywords:** motivation, learning, memory, instrumental, stimulus control

Pavlovian-instrumental transfer (PIT) examines how action learning (i.e., instrumental) and stimulus learning (i.e., Pavlovian) are integrated to control ongoing behaviors. It shows that this control can take at least two distinct forms (Holmes et al., 2010; Cartoni et al., 2016). In general PIT, a stimulus predicting an outcome of a particular motivational domain (i.e., appetitive or aversive) is shown to energize performance of actions associated with outcomes belonging to the same motivational domain. In specific PIT, a stimulus predicting a particular outcome is found to guide choice toward actions earning the same outcome and away from actions associated with different outcomes, even though these outcomes belong to the same motivational domain. PIT can therefore reflect a simple modulation of action performance (general PIT) or a more complex demonstrations of sensory-driven action selection (specific PIT). Although much progress has been made in describing the mechanisms underlying the two forms of PIT (Cartoni et al., 2016; Corbit and Balleine, 2016; Laurent and Balleine, 2011), much remains to be understood at both the psychological and neural level. The empirical and theoretical papers included in this Research Topic aimed to address this gap in knowledge and to demonstrate how PIT can provide insights into dysregulation and maladaptive behaviors.

A set of empirical papers used rodents to elucidate the psychological mechanisms and neural architecture underlying general and specific PIT. Specifically, Lingawi et al. manipulated the value of appetitive food outcomes to dissociate the control exerted by a predictive stimulus on action performance and action selection. They found that lowering outcome value abolished general PIT but left specific PIT mostly intact. However, Panayi and Killcross revealed that the latter can be disrupted when the manipulation lowering food value also reduces the capacity of a stimulus to retrieve information about its predicted outcome. Two other papers provide novel information about the neural circuity underlying general PIT (Halbout et al.) show that general PIT does not normally necessitate activity in the dorsomedial prefrontal cortex (dmPFC). However, they also show that the effect be blunted when dmPFC activity is artificially increased. In the other paper, Ge and Balleine provide the first evidence of the critical role played by the bed nucleus of stria terminalis (BNST) in supporting general PIT. The authors also offer an elegant model describing how the BNST may interact with other brain regions to achieve such function. Finally, Kim et al. demonstrates for the first time how general and specific PIT can be observed in the aversive domain and reveal their reliance on activity in the central amygdala.

In addition to rodent studies that attempt to dissect transfer effects from the psychological and neural perspective, our Research Topic includes work that explores the translation of PIT to humans in ways that relate to both basic and applied research. For example, a paper reporting an analysis of computational and representational elements that drive PIT in humans (Degni et al.) established procedures that isolate different forms of the transfer effect. Moreover, this study showed that general motivation effects are obtained under a variety of associative conditions. Non-specific, stimulus-enhanced responding was obtained when the outcome was either previously associated with an action, or not.

Other papers then underscore how PIT can provide insight into maladaptive behaviors and offer pathways to treatment. These include a rodent study exploring factors that influence craving for alcohol cues (Ginsburg et al.). This experiment used stimuli of varying length to show that cue duration inversely influences CS-elicited food-responding, while augmenting the transfer effect when longer duration ethanol-paired cues are tested. These data have profound implications for how we understand craving and provide an information processing framework from which to approach the issue.

The work reported here also includes a series of innovative studies showing how PIT can be used to investigate human disorders that directly or indirectly relate to emotion, including attention-deficit/hyperactivity (Geurts, den Ouden et al.), borderline personality (Geurts, Van den Heuvel et al.), and psychopathy (Geurts, von Borries et al.). In these studies, PIT was used to establish the efficacy of therapeutic treatments in patients with borderline disorder, while also providing psychopathy indicators in violent criminals. In studies examining ADHD patients across disorder subtypes, different degrees of inhibitory stimulus control were identified, and these differences were eliminated by combining traditional therapeutic approaches with cognition-based treatments.

This Research Topic convincingly demonstrates how PIT can be used to uncover the dynamics of different forms of learning and motivated behavior. It extends the utilization of the PIT task from a general exploratory task toward understanding the nature of dysregulation that underlies human disorder and the efficacy of relevant treatments. Together the studies reported in this collection provide new insights into the psychological and neural mechanisms supporting this intriguing phenomenon as well as these novel applications. However, these papers also underscore that much more work is required to obtain a clear understanding of the psychological and neural mechanisms that regulate PIT. Completing this work will be critical and there is great potential for the PIT task to provide increased sensitivity and subtlety to analyses in new and exciting areas across psychology and neuroscience. This will include the continued exploration of how PIT emerges from reward and other appetitive processes but also translating the task into new areas of motivation to identify parallels and idiosyncrasies.

## Author contributions

VL and VC wrote the editorial letter, organized, and edited the submissions. All authors contributed to the article and approved the submitted version.
